# Non-Vitamin K Antagonist Oral Anticoagulants and the Treatment of Venous Thromboembolism in Cancer Patients: A Semi Systematic Review and Meta-Analysis of Safety and Efficacy Outcomes

**DOI:** 10.1371/journal.pone.0114445

**Published:** 2014-12-05

**Authors:** Torben Bjerregaard Larsen, Peter Brønnum Nielsen, Flemming Skjøth, Lars Hvilsted Rasmussen, Gregory Y. H. Lip

**Affiliations:** 1 Department of Cardiology, Aalborg University Hospital, Aalborg, Denmark; 2 Aalborg Thrombosis Research Unit, Department of Clinical Medicine, Faculty of Health, Aalborg University, Aalborg, Denmark; 3 University of Birmingham Centre for Cardiovascular Sciences, City Hospital, Birmingham, United Kingdom; Kurume University School of Medicine, Japan

## Abstract

**Background:**

This study sought to investigate the relative efficacy and safety of non-vitamin K oral anticoagulants (NOACs) for the treatment of venous thromboembolism (VTE) in cancer patients.

**Methods:**

A systematic search of the PubMed, EMBASE, and ClinicalTrials.gov databases identified all multicentre, randomised phase III trials investigating the initial use of NOAC against a vitamin K antagonist (VKA) together with subcutaneous heparin or low molecular weight heparin (upstart) for treatment of VTE. Outcomes of interest were recurrent VTE (deep venous thrombosis or pulmonary embolism), and clinically relevant bleeding.

**Results:**

Four randomised controlled phase III trials were included, comprising a total of 19,060 patients randomised to either NOAC or VKA. For patients with active cancer (N = 759), the analysis on the efficacy outcomes demonstrated a trend in favour of NOAC (OR 0.56, 95% CI 0.28–1.13). Similar, analyses on the safety outcomes comparing NOAC to VKA and enoxaparin demonstrated a trend in favour of NOAC (OR 0.88, 95% CI 0.57–1.35).

**Conclusion:**

Point estimates of the effect size suggest an important estimated beneficial effect of NOAC in the treatment of VTE in cancer, in terms of efficacy and safety, but given the small numbers of patients with cancer in the randomised trials, statistical significance was not achieved.

## Introduction

Venous thromboembolism (VTE), including deep venous thrombosis (DVT) and pulmonary embolism (PE), is a major healthcare concern that results in considerable long-term morbidity and mortality and affects more than 1.6 million individuals each year across the United States and the European Union [1]–. Patients with symptomatic VTE have a high and persistent risk of recurrent events, including non-fatal and fatal PE [4]. Estimates suggest a cumulative incidence of recurrent VTE from 17.5 percent after 2 years of follow-up increasing to more than 30 percent after 8 years [5], [6].

The association of VTE with cancer is well known and has been described in large cohort studies [7], [8]. Cancer combined with VTE is associated with a poor outcome in terms of recurrent thrombosis and survival [9]–[11]. Despite vitamin K antagonist (VKA) therapy, cancer patients have twice as many relapses and 3 times as many bleeding cases as non-cancer patients in spite of careful treatment control with frequent INR measurements [12]. Other challenges are the increased comorbidity, multi pharmacological treatment with potential interactions and the resulting difficulty in controlling INR, resulting in poor quality anticoagulation control, as reflected by reduced time in therapeutic range, that has implications for the efficacy and safety of the VKAs [13], [14]. In cancer patients, INRs may also be affected by nausea, for example in conjunction with chemotherapy. Furthermore, invasive procedures as part of the investigation or treatment of cancer, such as chemotherapy, increase the risk of complications and are likely to cause thrombocytopenia and other serious side effects. This can lead to the need for delayed or reduced dosing in VKA therapy with implication of efficacy of the anti-thrombotic treatment.

Standard treatment for VTE has been the administration of heparin or low molecular heparin (LMWH), overlapped and followed by a vitamin K antagonist [15]. This standard regimen is effective but complex, especially in patients with cancer who are challenged by intensive surgical and medical therapy and by having periods of their disease characterized by changing appetite and food intake. To overcome some of these challenges, the first large multicentre, randomised, open-label clinical trial was performed to investigate whether LMWH (dalteparin) was more effective and safer than oral anticoagulant therapy in preventing recurrent VTE in patients with cancer who have acute VTE [16]. This study showed that dalteparin was more effective than an oral anticoagulant in reducing the risk of recurrent thromboembolism without increasing the risk of bleeding. Non-vitamin K antagonist oral anticoagulants (NOACs, previously referred to as new or novel oral anticoagulants [17]) directed against factor Xa or thrombin overcome some limitations of standard therapy, including the need for injection and for regular dose adjustments on the basis of laboratory monitoring [18]–[22]. The clinical trials investigating the effects of the NOAC's were not aimed at patients with VTE and cancer, although these patients were not excluded in the majority of the studies.

Treatment with a NOAC would be an attractive alternative to either the standard VKA treatment or injection treatment, but it is unknown whether this therapy is effective and safe. The purpose of this meta-analysis is to examine the NOAC as an alternative to standard treatment with VKA and LMWH in patients with VTE and cancer.

## Methods

The methods applied in this study are consistent with those proposed in the Preferred Reporting Items for Systemic Reviews and Meta-Analyses (PRISMA) statement [23].

### Study selection

We searched Medline and EMBASE from Jan 1, 2009 to Apr 02, 2014 and conducted a semi-systematic review. MeSH terms as “venous thromboembolism” and “warfarin” and (“dabigatran” or “rivaroxaban” or “apixaban” or “edoxaban” or “oral factor Xa inhibitor” or “oral thrombin inhibitor”) were used. We also did a search of ClinicalTrials.gov to identify relevant ongoing clinical studies. The population, intervention, comparison, outcome, and study design (PICOS) [24] of eligible trials for the meta-analysis were specified as follows: patients with acute symptomatic VTE as indication for treatment were eligible for inclusion. Intervention of interest was primary anticoagulant treatment of patients with NOAC and compared to treatment with VKA. Further, the trials should report both efficacy and safety outcomes according to cancer history status. We only included phase III, randomised controlled trials in this study. Outcomes of interest included recurrent VTE and clinically relevant bleeding, see online supporting informaion for details. Despite the risk of bias, open-label and blinded studies were allowed since VKA requires monitoring for dose adjustment, which makes blinding a challenge. Search strings and PICOS are provided in Table S1 in File S1.

### Assessment of study eligibility and inclusion

All titles and abstracts identified through the initial search in the digital literature were reviewed independently by two reviewers (TBL, PBN), and any disagreements were resolved by discussion and/or referral to a third reviewer (GYL) as necessary. Articles obviously irrelevant on the basis of title and abstract were excluded. Eligibility of the remaining articles was assessed on the basis of the inclusion criteria listed above. When there was more than one unique article for a given study, the articles were grouped together for reviewing purposes. Systematic reviews and meta-analyses that meet the inclusion criteria were not reviewed but were used to identify any other potentially relevant articles.

The methodological quality of the included studies was evaluated in accordance with the Cochrane collaboration's risk of bias tool [25] on the basis of the randomisation process, allocation concealment (adequate, unclear, inadequate, or not used), degree of blinding, particularly of the outcome assessors and patient attrition rate.

### Data extraction

Information regarding study design (including intervention/comparators) and characteristics of study participants was extracted: study design, sample size, patient inclusion and exclusion criteria, patient characteristics (e.g. mean/median age, gender, follow-up time, discontinuation rates, and co-morbidities); primary and secondary outcome measures, length of follow-up, statistical methods employed, effect sizes and uncertainty, and time in therapeutic range (TTR) for those receiving VKA treatment. The main efficacy outcome was a composite endpoint of recurrent VTE and the main safety outcome was clinically relevant bleeding defined as both minor and major clinically relevant bleeding events.

We sought to only include data from the intention-to-treat analysis of each study for the efficacy outcome in order to preserve the bias reduction from the RCT study design; whereas the per-protocol analysis was preferred for the safety outcome analysis. In case of double reporting of the same patient populations, data from the main publication was extracted. If subgroup analyses were reported in additional articles, this information was included and supplemented by information from other publications if appropriate.

### Statistical analysis and synthesis

Our main focus was to investigate if patients could benefit from NOAC compared to VKA treatment, in terms of efficacy and safety outcome.

Pooled odds ratios (OR) and 95% confidence intervals (CI) in strata based on baseline status for cancer were estimated using the DerSimonian and Laird random-effects models to account for within- and between study variations [26]. Study outcomes were based on reported number of events and population size. The number of events was weighted by the inverse follow-up time to account for possible varying time of follow-up between studies assuming constant hazard rate. Sensitivity analysis without weighting was performed. Study heterogeneity was assessed by the I^2^ statistics. A P-value<0.05 was considered statistically significant. STATA version 12.1 (Stata Corporation, College Station, TX) was used for statistical analyses and graphical presentations.

## Results

### Study selection and characteristics

**Figure 1:**
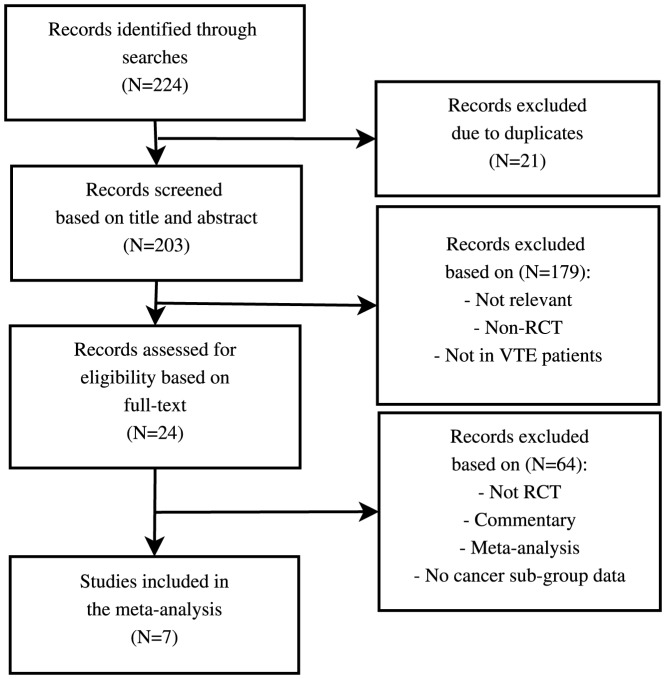
Flowchart of the study selection process. RCT: Randomised controlled trial. VTE: Venous thromboembolism. PE: pulmonary embolism.

The electronic search identified 203 records with no duplicates (see Figure 1). Based on screening of title and abstract 179 of 203 (88%) records were removed, and 24 full-text papers were retrieved and evaluated for eligibility. Of these 9 papers fulfilled the PICOS inclusion criteria. Of the 5 [18]–[22] RCTs we identified 4 studies that had sufficient data to perform subgroup analyses based on cancer status (no sub-group data available from the AMPLIFY study investigating apixaban [22]). The included trials investigated the efficacy and safety of NOACs in either DVT or PE. For safety, all four studies followed the definition of major bleeding in clinical investigations of anti-haemostatic medicinal products in non-surgical patients [27]. An overview of selected study characteristics are provided in Table 1.

**Table 1:** Selected patient characteristics of the included randomized trials.

Characteristics	EINSTEIN DVT (n = 4,832)		EINSTEIN PE (n = 3,449)		RE-COVER (n = 2,539)		Hokusai-VTE (n = 8,240)	
	NOAC	Standard	NOAC	Standard	NOAC	Standard	NOAC	Standard
Mean age - yr	57.9	57.5	55.8	56.4	55.0	54.4	55.7	55.9
Male sex - %	54.1	51.7	57.4	56.3	58.0	58.9	57.3	57.2
Follow-up - months	3, 6 and 12 months		3, 6 and 12 months		6 months		12 months	
NOAC	Rivaroxaban 15 BID for 3 weeks, followed by 20 mg OD		Rivaroxaban 15 BID for 3 weeks, followed by 20 mg OD		Dabigatran 150 mg BID		Edoxaban 60 mg OD	
Randomized study design	Open-label, assessor-blind, non-inferiority study of efficacy for 3, 6 or 12 months treatment for of DVT without symptomatic PE		Open-label, assessor-blind, non-inferiority study of efficacy for 3, 6 or 12 months treatment of PE with or without symptomatic DVT		Double Blind, parallel-group study of efficacy and safety for 6 months treatment of acute symptomatic VTE		†Open-label, parallel-group study of efficacy and safety for a maximum of 12 months treatment of symptomatic DVT and/or PE.	
Inclusion criteria	Acute symptomatic proximal DVT, no symptomatic PE		Symptomatic PE, no acute symptomatic proximal DVT		Acute, symptomatic, objectively verified proximal DVT of the legs or PE and for whom 6 months of anticoagulant therapy was considered to be an appropriate treatment were potentially eligible		Acute symptomatic proximal DVT and/or symptomatic PE confirmed at the site by appropriate diagnostic imaging	
Exclusion criteria	Allows: 100 mg aspirin and 75 mg clopidogrel (dual antiplatelet treatment)				Allows: 100 mg aspirin		Dual antiplatelet treatment	
	Hypertension >180/110 mmHg				Life expectancy of less than 6 months		Uncontrolled hypertension: >170/100 mmHg despite treatment	
	Life expectancy less than 3 months				Recent unstable cardiovascular disease		Active cancer where indication of long-term LMWH is anticipated	
							Life expectancy less than 3 months	
Dose Adjustments	First 3 weeks 15 mg, followed by 20 mg				No dose adjustments		30 mg if: 1) Body weight ≤60 Kg, 2) Creatine clearance of 30–50 ml/min, 3) concomitant use of the P-glycoprotein inhibitors verapamil or quinidine	
Year of publication	2010		2012		2009		2013	
Primary efficacy outcome	Symptomatic recurrent VTE		Symptomatic recurrent VTE		Symptomatic recurrent VTE		Symptomatic recurrent VTE	
Main safety outcome	Clinically relevant bleeding, defined as the composite of major or clinically relevant nonmajor bleeding		Clinically relevant bleeding, defined as the composite of major or clinically relevant nonmajor bleeding		Clinically relevant bleeding, defined as the composite of major or clinically relevant nonmajor bleeding		Clinically relevant bleeding, defined as the composite of major or clinically relevant nonmajor bleeding	
Cancer, %	4.7		4.5		6.8		5.2	
Creatinine clearance <50 ml/min, %	8.8	7.9	6.9	7.5	5.0	4.5	9.2	9.5
Time in therapeutic range - %	NA	62.7	NA	58.0	NA	60	NA	‡63.5

NOAC: non-vitamin K oral anticoagulant. DVT: deep venous thrombosis. PE: pulmonary embolism. VTE: venous thromboembolism. OD: once daily. BID: twice daily. NA: not available

† In contrast to the three other studies which used a single-drug approach for all treatment phases, the Hokusai-VTE used a traditional sequence of a heparin lead-in followed by an oral agent.

‡ In the Hokusai-VTE study the time in the therapeutic INR range was calculated excluding the initial heparin lead-in period and with correction for planned interruptions.

The trials included a total of 19,060 patients, of these 4% (N = 759, 405 and 354 in the NOAC and VKA group, respectively) had active cancer at inclusion. Events and rates at 12 months follow-up for each trial by cancer status are reported in Table 2. The RE-COVER study [18] did not report major bleeding and recurrent VTE were reported at 6 months follow-up.

**Table 2:** Clinical outcomes in acute treatment of venous thromboembolism in patients with and without cancer.

Efficacy end point events, recurrent venous thromboembolism Intension to treat populations, 12 months follow-up					
Study	Active cancer at baseline	NOAC		Standard therapy	
		Events, % (N)	Total, N	Events, % (N)	Total, N
Einstein DVT	Yes	3.4 (4)	118	5.6 (5)	89
	No	2.0 (32)	1613	2.8 (46)	1629
Einstein PE	Yes	1.8 (2)	114	2.8 (3)	109
	No	2.1 (48)	2305	1.8 (41)	2304
RE-COVER*	Yes	3.1 (2)	64	5.3 (3)	57
	No	2.3 (28)	1209	2.0 (24)	1209
Hokusai-VTE	Yes	3.7 (4)	109	7.1 (7)	99
	No	3.1 (126)	4009	3.5 (139)	4023
**Summary**			**9541**		**9619**

Safety end point events, major bleeding Per-protocol populations, 12 months follow-up					
Study	Active cancer at baseline	NOAC		Standard therapy	
		Events, % (N)	Total, N	Events, % (N)	Total, N
Einstein DVT	Yes	14.4 (17)	118	15.9 (14)	88
	No	7.6 (122)	1600	7.6 (124)	1623
Einstein PE	Yes	12.3 (14)	114	9.3 (10)	108
	No	10.2 (235)	2298	11.5 (264)	2297
RE-COVER	Yes	NA	NA	NA	NA
	No	NA	NA	NA	NA
Hokusai-VTE	Yes	18.3 (20)	109	25.3 (25)	99
	No	8.2 (329)	4009	9.9 (398)	4023
**Summary**			**8248**		**8338**

NOAC: Non-vitamin K oral anticoagulant. NA: Not available

*Reported events at 6 months follow-up.

### Results from meta-analysis

**Figure 2:**
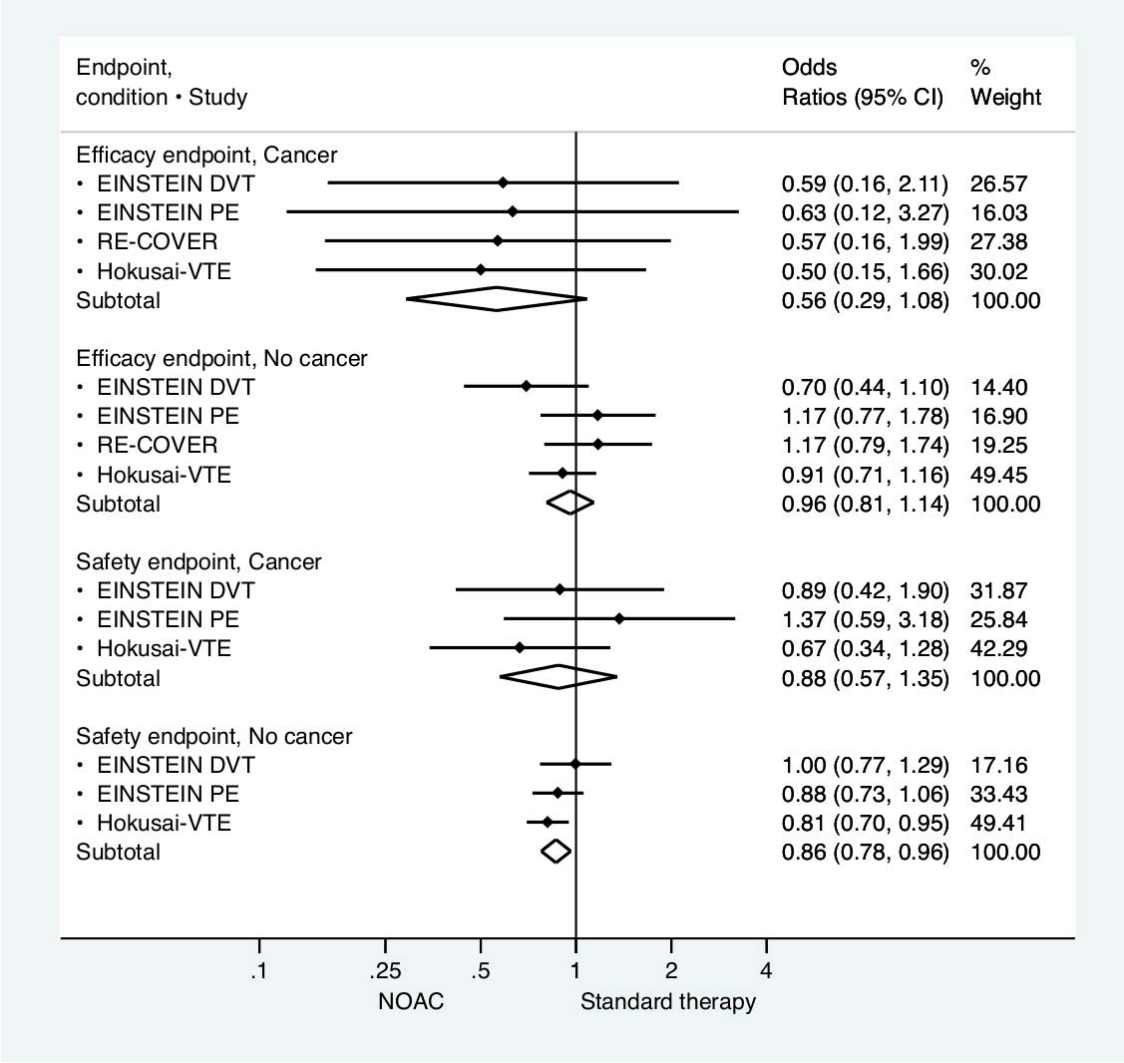
Study level and weighted across study efficacy and safety of non-vitamin K oral anticoagulants in treatment of acute venous thromboembolism in patients with and without cancer. CI: confidence interval. NOAC: Non-vitamin K oral anticoagulation.

Randomization to NOACs showed a non-significant trend towards higher efficacy for patients who had active cancer at inclusion compared to VKA (OR 0.56, 95% CI 0.28, 1.13), see Figure 2. The analysis demonstrated similar non-significant result favouring NOACs for VKA regarding the overall safety outcomes (OR 0.88, 95% (0.57, 1.35). Indication of between-study heterogeneity was only seen for efficacy in the no-cancer subgroup (I^2^ statistics  = 25.7%). Test for interaction between the cancer vs non-cancer group was not non-significant (p = 0.65), notwithstanding that the two populations (despite both included in the trials) were clinically different in both baseline characteristics and in respond patterns to antithrombotic therapy. Funnel plots and tests for small sample bias are provided in the Figure S1 in File S1.

### Quality assessment

Quality assessment was undertaken with the Cochrane tool as a guide for risk bias assessment [25] (provided in Table S2 in File S1). Some aspects of the study designs and analyses could contribute to bias.

The EINSTEIN-DVT [19] and EINSTEIN-PE [20] (rivaroxaban) used per-protocol analysis for endpoints, but the intention-to-treat analysis was provided on the primary efficacy endpoint.

The RE-COVER study [18] used a modified intention-to-treat analysis, where patients in the intervention-arm who did not receive dabigatran were excluded from all analysis; safety was analysed in the per-protocol cohort. Further, as specified in the protocol, all patients who prematurely stopped (adverse event or non-adherent) receiving treatment with dabigatran were intended to be followed for 6 months, but this was not always fulfilled.

In the Edoxaban Hokusai-VTE Study [21] the primary efficacy outcome was analysed by a modified intention-to-treat method, which included patients who underwent randomisation and received at least one dose of edoxaban. The period used for this analysis was the time during which the patients were receiving the study drug or within 3 days after the last dose of edoxaban was stopped or interrupted.

## Discussion

This meta-analysis is the first study to systematically include results from three NOACs studied in the key phase III clinical trials for treatment of patients with acute VTE and cancer. Our results, based on 759 patients with cancer and acute VTE, showed that recurrent VTE and bleeding events were reduced in patients receiving NOACs as acute treatment for VTE. However, given the small numbers of patients with cancer in the randomised trials, statistical significance was not achieved despite point estimates suggest an important estimated beneficial effect of NOACs in the treatment of VTE in cancer, for both efficacy and safety.

Several open-label, randomized controlled trials have provided evidence of improved efficacy and safety of LMWH vs. VKA in the prevention of recurrent VTE in patients with cancer-associated VTE [28]–[31]. The largest of these trials, the CLOT trial (Randomized Comparison of Low-Molecular-Weight Heparin versus Oral Anticoagulant Therapy for the Prevention of Recurrent Venous Thromboembolism in Patients with Cancer), included 672 patients with 6-months follow-up [28]. A subsequent meta-analysis have confirmed the observations on efficacy and safety, reporting a relative risk reduction of recurrent VTE of more than fifty percent, but with no significant reduction in death [32].

Treatment of patients with VTE and cancer is a challenge because of the many conditions that affect both the patient and the different treatment modalities that often accompanies the disease, such as surgery and pharmacological treatment. This calls for drugs with a short half-life and easy administration which is largely met by NOACs. However, even if NOACs will be tested in cancer patients, this will probably not solve all challenges in these patients. Interactions between chemotherapeutic agents and immunosuppressant's with NOACs are still possible, based on known metabolic pathway activities. Drugs that inhibit P-glycoprotein transport or CYP3A4 pathway (for example, cyclosporine and tamoxifen) can increase NOAC levels, and drugs that induce P-glycoprotein transport or CYP3A4 pathway can lower NOAC levels (for example, dexamethasone, doxorubicin, and vinblastine). Cancer patients treated for systemic fungus infection could be another challenge, as the antifungal azoles could increase the effect of some NOACs (the azoles are strong P-glycoprotein transport inhibitors).

### Limitations

The present meta-analysis assumes that all the NOACs are the same (which they are not) and work on the basis of a class effect or are broadly equivalent; and that the randomised trials are homogeneous, which again they are not. Furthermore, the designs of the studies were not similar, with different follow-up times, and different time in therapeutic range. However, the studies included, were similar, not only on baseline characteristics (Table 1), but also on the outcomes reported, and were all tested against warfarin. In none of the studies, specific definitions of cancer were provided. It remains unknown if the type or the location of cancer would change the effect from NOACs, and it is not clear if the bias identified in the studies might impact the findings. Indeed, the relative efficacy and safety of NOACs was generally consistent, although EINSTEIN-PE favoured standard therapy compared to a NOAC (rivaroxaban) in patients with active cancer. We emphasise that caution is warranted when interpreting results from subgroup analyses obtained from studies not designed (or powered) to the conducted subgroup analyses.

In conclusion, point estimates of the effect size suggest an estimated beneficial effect of NOACs in the treatment of VTE in cancer, in terms of efficacy and safety - but given the small numbers of patients with cancer in the randomised trials, statistical significance was not achieved. The relative efficacy and safety of NOACs was consistent across most studies. Our findings call for larger studies on NOACs as a therapeutic option for patients with VTE and cancer.

## Supporting Information

File S1 **Supporting tables and figures.** **Table S1**. Search strategy. **Table S2**. Risk of bias. **Figure S1**. Funnel plots.
(DOCX)

Checklist S1 **PRISMA checklist.**
(DOC)
