# Supplementary material for: Non-Vitamin K Antagonist Oral Anticoagulants and the Treatment of Venous Thromboembolism in Cancer Patients: A Semi Systematic Review and Meta-Analysis of Safety and Efficacy Outcomes
Source: PLoS One. 2014 Dec 5;9(12):e114445. doi: 10.1371/journal.pone.0114445 (PMC4257692; doi:10.1371/journal.pone.0114445)
Supplement: File S1 — Supporting tables and figures. Table S1. Search strategy. Table S2. Risk of bias. Figure S1. Funnel plots. (DOCX) [file pone.0114445.s001.docx]

**Supplementary Material**

**Non-vitamin K antagonist oral anticoagulants and the treatment of venous thromboembolism in cancer patients:**

**A semi systematic review and meta-analysis of safety and efficacy outcomes.**

Torben Bjerregaard Larsen^1, 2^

Peter Brønnum Nielsen^1, 2^

Flemming Skjøth^1, 2^

Lars Hvilsted Rasmussen^2^

Gregory Y. H. Lip^2, 3^

1. Department of Cardiology, Aalborg University Hospital, Denmark

2. Aalborg Thrombosis Research Unit, Department of Clinical Medicine, Faculty of Health, Aalborg University, Aalborg, Denmark.

3. University of Birmingham, Centre for Cardiovascular Sciences, City Hospital, Birmingham, United Kingdom

**Table S1:** Search strategy

**PICOS:**

PICOS for meta-analysis of NOAC’s and cancer

Population: Patients receiving OAC for acute, symptomatic deep-vein thrombosis and/or acute, symptomatic pulmonary embolism

Intervention: Novel anticoagulant; dabigatran or rivaroxaban or apixaban or edoxaban

Comparator: Standard vitamin K-antagonist treatment (INR range 2-3) including use of low molecular weight heparin

Outcome: Stroke or systemic embolism, major bleeding, intracranial haemorrhagic, ischaemic stroke, haemorrhagic stroke, all-cause death, stratified by active cancer yes/no

Study design: Randomized controlled trials

Other: No language restrictions were made.

**PubMed:**

("Venous Thromboembolism"[Mesh] OR Venous Thromboembolism[Title/Abstract] OR "pulmonary embolism"[Mesh] OR "pulmonary embolism"[Title/Abstract] ) AND ("Warfarin"[Mesh] OR "Warfarin"[Title/Abstract] OR “Vitamin K antagonist”[Title/Abstract] ) AND ("dabigatran" [Title/Abstract] OR "dabigatran etexilate" [Title/Abstract] OR "apixaban"[Title/Abstract] OR "rivaroxaban" [Title/Abstract] OR "edoxaban" [Title/Abstract] OR “oral thrombin inhibitor”[Title/Abstract])

Limits: Humans

**EMBASE:**

((“Warfarin” OR “Vitamin K antagonist”) and ("Pulmonary embolism" or "Venous Thromboembolism") and ("dabigatran" or "apixaban" or "rivaroxaban" or "edoxaban")).

Limits: Human, EMBASE, Randomize controlled trial

**Results:**

Pubmed: 184

EMBASE: 40

Duplicates: 21

Resulting in 203 records

| **Table S2:** Risk of bias |  |  |  |  |
| --- | --- | --- | --- | --- |
| **Cochrane tool for assessing risk of bias** | **EINSTEIN DVT** | **EINSTEIN PE** | **RE-COVER** | **Hokusai-VTE** |
| **Allocation** | Clear | Clear | Clear | Clear |
|  |  |  |  |  |
| **Blinding** | Clear | Clear | Clear | Clear |
|  |  |  |  |  |
| **Incomplete outcome data** | Clear | Clear | Clear | Clear |
|  |  |  |  |  |
| **Selective reporting** | Safety outcomes analysed on patients who received at least one study-drug | Safety outcomes analysed on patients who received at least one study-drug | We analysed efficacy according to a modified intention-to-treat principle, since patients who did not receive any study drug were excluded from all analyses, as was pre-specified in the protocol. For safety analyses, including bleeding episodes, events were considered from the time of the first intake of the study drug to 6 days after the last intake of the study drug; these analyses were performed on the basis of the patient’s actual treatment with the study drug. | 1) Modified intention-to-treat population, which included all patients who underwent randomization and received at least one dose of the study drug.  2) The primary efficacy outcome was evaluated for the on-treatment period — the time during which the patients were receiving the study drug or within 3 days after the study drug was stopped or interrupted.  3) Safety outcomes analysed per-protocol |
|  |  |  |  |  |
| **Other potential sources of bias** | None | None | Although it was intended that all patients who stopped the study drug owing to an adverse event or who were considered to show non-adherence would complete 6 months of follow-up, this did not always occur | None |
|  |  |  |  |  |

**Figure S1**

Funnel plot of included studies for efficacy and safety endpoint according to cancer subgroup. Vertical dashed line indicate group mean log OR.

Test for small study effects (p-value):

Eggers test Peter’s test

Efficacy endpoint, Cancer 0.359 0.976

Efficacy endpoint, No cancer 0.755 0.869

Safety endpoint, Cancer 0.100 0.370

Safety endpoint, No cancer 0.047 0.108
